# Thrombosed Popliteal Venous Aneurysm Treated With a Great Saphenous Vein Roll Interposition Technique

**DOI:** 10.7759/cureus.81199

**Published:** 2025-03-25

**Authors:** Yoshun Sai, Hiroki Sakai, Kunihiko Yoshino, Joji Ito

**Affiliations:** 1 Cardiovascular Surgery, Tokyo Bay Urayasu Ichikawa Medical Center, Chiba, JPN

**Keywords:** great saphenous vein grafting, popliteal vein, pulmonary embolism (pe), vascular bypass, venous aneurysm

## Abstract

Popliteal venous aneurysms (PVAs) are rare vascular anomalies that can result in serious thromboembolic complications. We present a case of a 77-year-old woman with a thrombosed right PVAs complicated by pulmonary embolism. Following initial anticoagulation, surgical resection of the aneurysm was performed, and individualized venous reconstruction was achieved using an autologous great saphenous vein (GSV) roll graft. This technique was selected over conventional panel or spiral grafts due to its simplicity, adaptability to size discrepancy, and reduced suture lines. To our knowledge, the use of a GSV roll graft, specifically tailored for popliteal vein reconstruction in thrombosed PVAs, has not been previously reported. Postoperative anticoagulation was continued with direct oral anticoagulants. Graft patency was confirmed by duplex ultrasonography and contrast-enhanced CT venography at regular intervals. At 1.5 years post-surgery, graft patency was maintained, and the patient remained symptom-free. This case highlights the effectiveness of individualized venous reconstruction using a GSV roll interposition graft in managing complex thrombosed PVAs.

## Introduction

Popliteal venous aneurysms (PVAs) are uncommon vascular entities characterized by abnormal dilatation of the popliteal vein. Though relatively rare, PVAs carry significant clinical importance due to their association with potentially life-threatening thromboembolic complications, particularly pulmonary embolism (PE). The pathogenesis of PVAs remains incompletely understood, but factors such as venous wall degeneration, congenital abnormalities, inflammation, and trauma have been proposed. Clinically, PVAs may present asymptomatically or manifest through thrombotic complications, pain, swelling, or palpable masses behind the knee [1].

Given the substantial risk of recurrent thromboembolic events, despite anticoagulation alone, surgical intervention is widely considered essential, especially in symptomatic patients or those presenting with significant aneurysmal dilatation. A commonly accepted diagnostic criterion for PVA is a localized venous dilatation exceeding 50% of the native vein diameter or a popliteal vein diameter of ≥20 mm. Although universally established guidelines are lacking, surgical treatment is generally recommended for symptomatic PVAs or asymptomatic ones greater than 20 mm in diameter due to the risk of PE [2].

Traditional surgical techniques for managing PVAs, such as tangential aneurysmectomy with lateral venorrhaphy, are commonly utilized, but they may not be adequate for complex cases involving significant thrombus load or size discrepancies between graft and native vessels. In these scenarios, alternative strategies, including complete aneurysm resection and autologous graft reconstruction, may offer improved outcomes. Among available graft options, we selected a great saphenous vein (GSV) roll graft because it enables tailored adjustment to diameter discrepancies, reduces the number of suture lines compared to spiral grafts, and simplifies the technical procedure. This report describes a rare case of a large, thrombosed PVA managed successfully using this technique, highlighting its potential advantages in addressing complex anatomical challenges.

## Case presentation

A 77-year-old woman, with a medical history of breast cancer and osteoporosis, was brought to our hospital after experiencing a sudden loss of consciousness in the restroom, resulting in facial trauma. The syncopal episode was presumed to be secondary to acute pulmonary embolism, given the concurrent findings and hypoxemia; however, other potential causes such as vasovagal syncope or orthostatic hypotension could not be definitively excluded. On initial assessment, her Glasgow Coma Scale (GCS) score was E4V5M6, blood pressure was 119/83 mmHg, heart rate was 116 beats per minute, respiratory rate was 19 breaths per minute, oxygen saturation was 84% on room air, and body temperature was 36.7°C.

**Figure 1:**
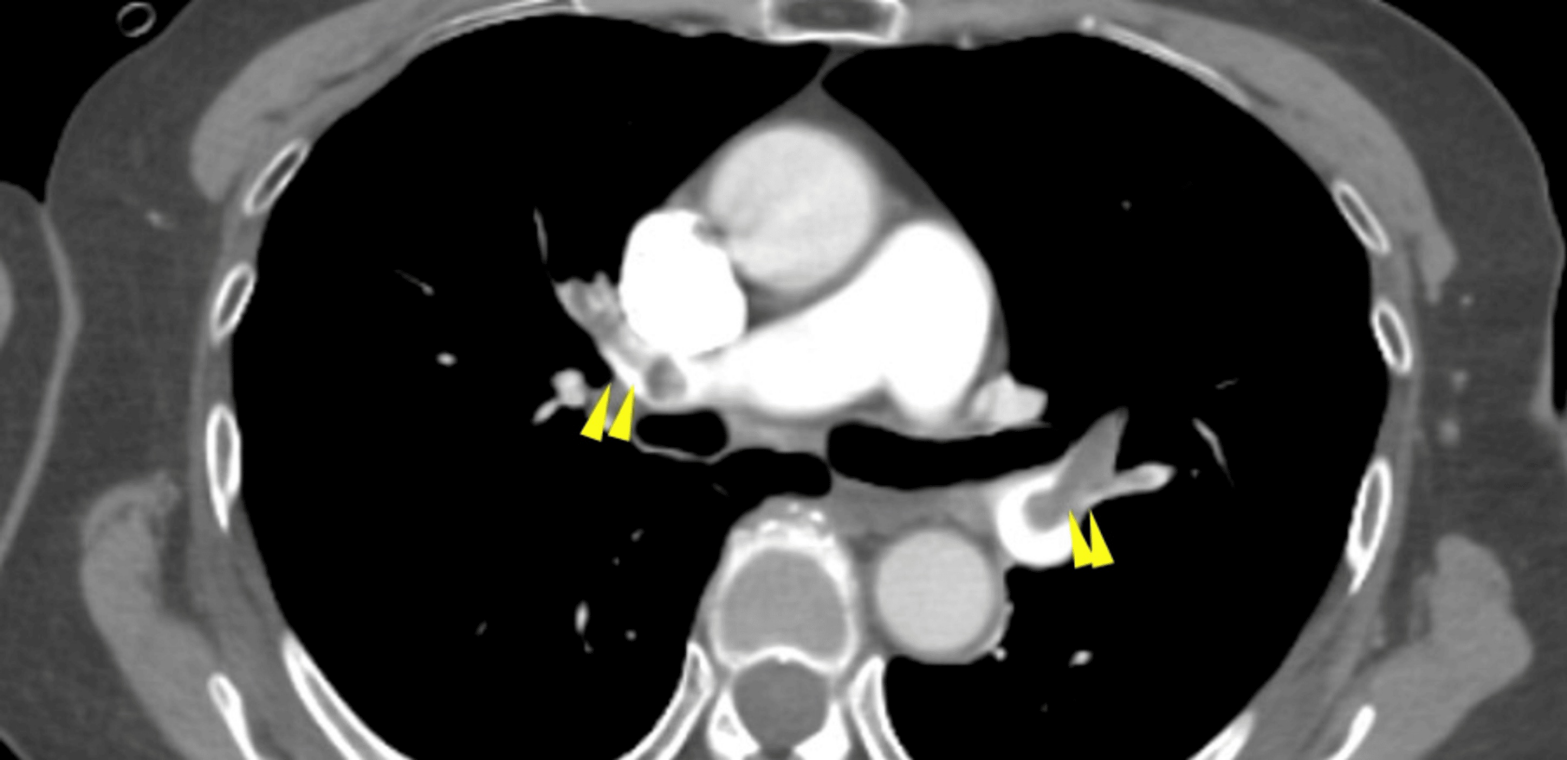
Contrast-enhanced CT revealed the presence of pulmonary artery thrombi (yellow arrowheads).

**Figure 2:**
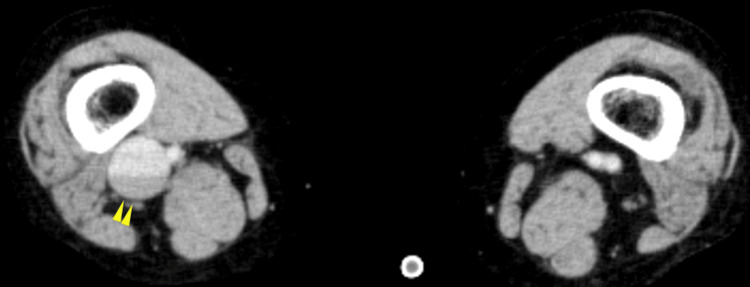
Contrast-enhanced CT revealed the presence of a right popliteal venous aneurysm (yellow arrowheads).

A 12-lead electrocardiogram (ECG) revealed sinus tachycardia with a heart rate of 103 beats per minute. Contrast-enhanced computed tomography (CT) identified thrombi within the bilateral pulmonary arteries, as well as a fusiform dilatation of the right popliteal vein measuring approximately 6 cm in length and 2.2 cm in diameter, consistent with a thrombosed popliteal venous aneurysm (Figures 1, 2).

Based on these findings, the patient was diagnosed with pulmonary embolism secondary to deep vein thrombosis originating from the aneurysm, and continuous intravenous heparin infusion was initiated.

On hospital day three, a follow-up contrast-enhanced CT demonstrated a reduction in pulmonary arterial thrombi, and transthoracic echocardiography revealed a decrease in the tricuspid regurgitation pressure gradient (TRPG) from 60 mmHg at admission to 40 mmHg. Given the substantial thrombus burden within the aneurysm, its large size, and the significant risk of recurrent thromboembolism, we elected to proceed with surgical intervention. Conservative management was initially considered but deemed inappropriate due to the presence of symptomatic PE. Among surgical options, complete resection with autologous graft reconstruction was selected over tangential aneurysmectomy or patch repair due to the need for removal of the entire thrombus-laden aneurysm and the goal of reducing recurrence.

**Figure 3:**
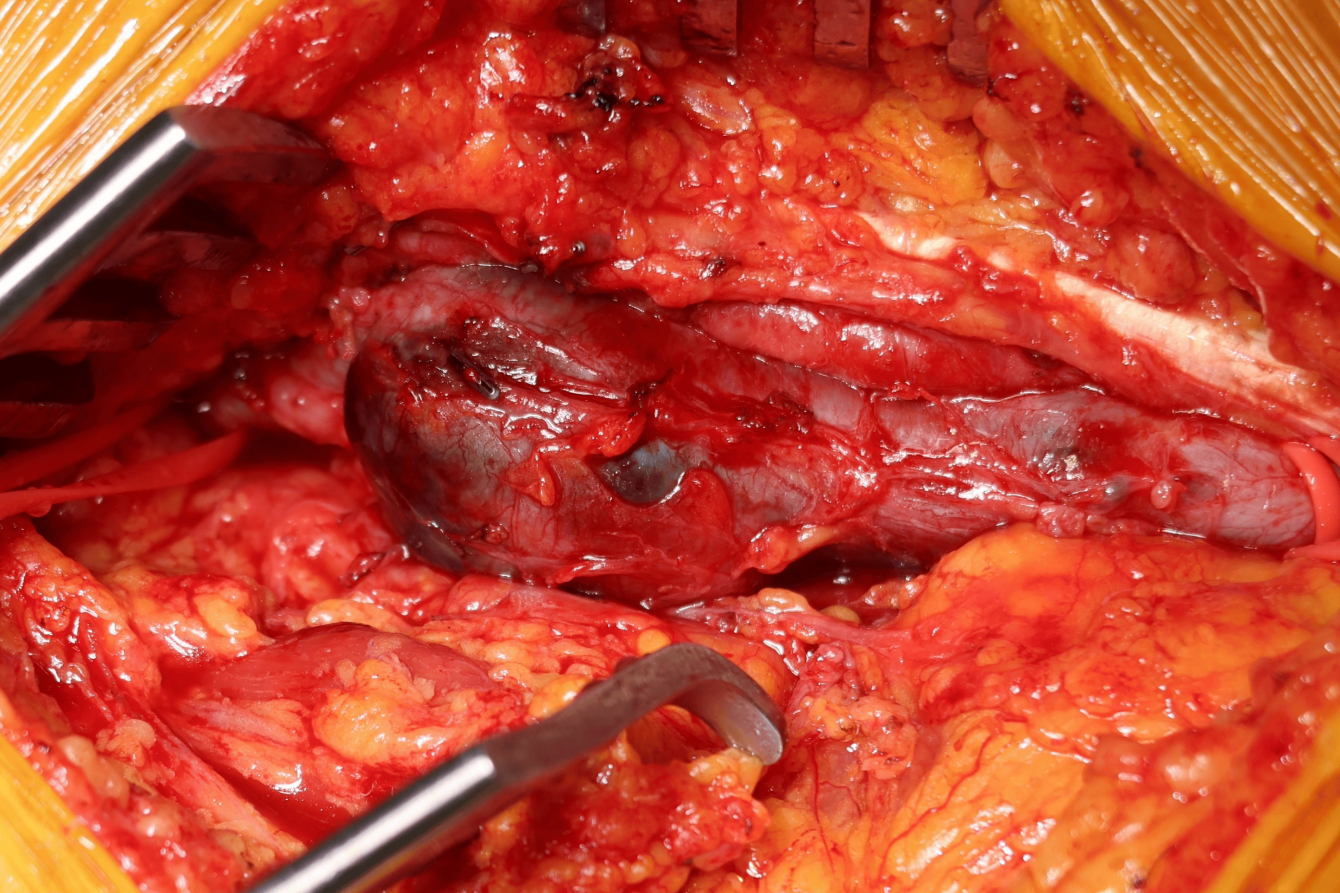
Intraoperative image of the popliteal venous aneurysm. The aneurysm was fusiform in shape, measuring approximately 6 cm in length and 2.2 cm in maximum diameter.

Under general anesthesia, the patient was placed in the supine position. A longitudinal incision, approximately 8 cm in length, was made along the medial aspect of the right thigh. This approach was selected because the aneurysm was located proximal to the knee joint, making it more accessible via a medial thigh incision rather than the conventional posterior S-shaped incision in the prone position. The popliteal venous aneurysm was identified intraoperatively as fusiform, measuring approximately 6 cm at its longest axis (Figure 3).

A 12 cm segment of the GSV was harvested from the left thigh. Due to a diameter mismatch - 6 mm for the native popliteal vein and 3 mm for the GSV - a composite graft was created by bisecting the harvested GSV and suturing the opened segments longitudinally in parallel. During graft preparation, all visible venous valves were carefully excised to prevent potential obstruction of venous flow in the reconstructed segment. This GSV roll graft technique was preferred over standard interposition grafting, cuff technique, or patch angioplasty because it allowed tailored adjustment of graft diameter, minimized suture lines, and provided a uniform conduit, thereby potentially improving long-term patency.

**Figure 4:**
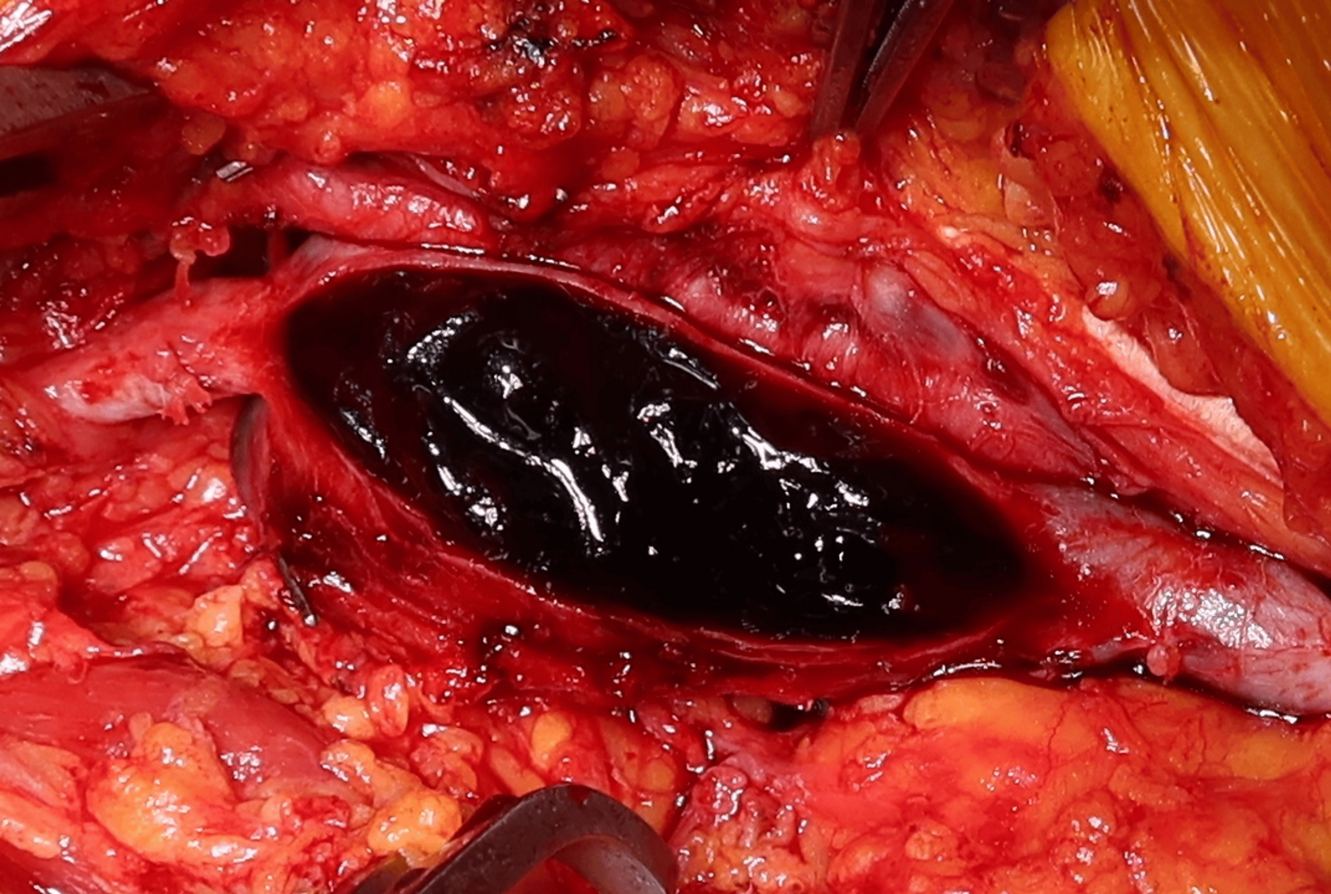
Intraoperative view of the opened aneurysmal sac. The lumen was filled with a large volume of red, organized thrombus, suggesting a chronic thrombotic process.

Systemic heparinization was administered prior to vessel clamping. After clamping the proximal and distal segments of the popliteal vein, the aneurysmal sac was incised, revealing a large volume of organized red thrombus (Figure 4).

**Figure 5:**
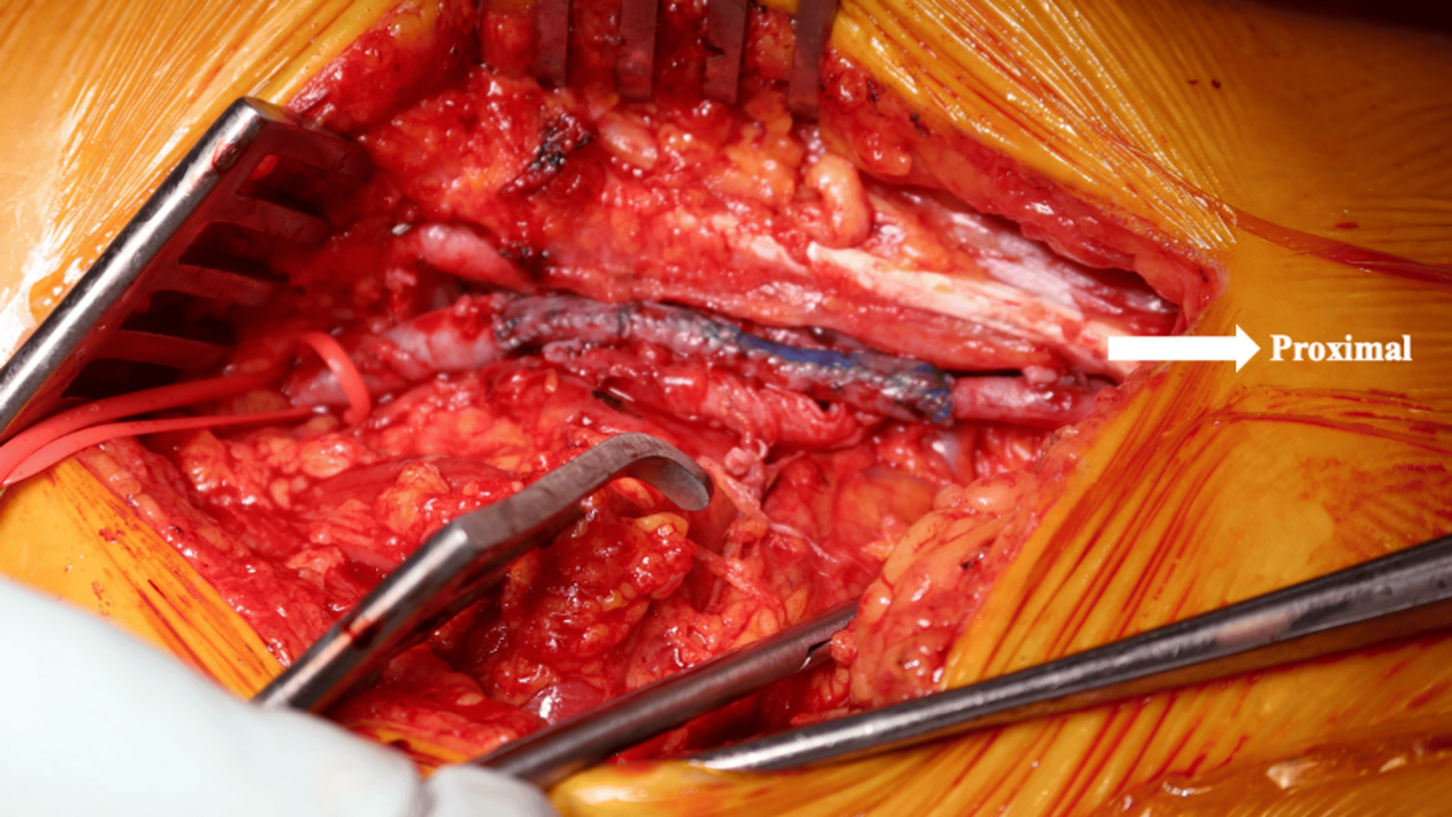
Intraoperative image after completion of vascular reconstruction. The custom-constructed great saphenous vein roll graft was anastomosed in an end-to-end fashion. The graft was oriented to follow the natural direction of venous flow from distal to proximal (white arrowhead).

Complete thrombus evacuation and resection of the aneurysmal wall were performed. The custom-constructed GSV roll graft was then anastomosed in an end-to-end fashion using 7-0 monofilament sutures (Figure 5). Intraoperative ultrasound confirmed satisfactory venous flow following declamping.

Postoperatively, intravenous heparin was continued for three days and then transitioned to edoxaban (30 mg/day), selected based on its favorable safety profile and once-daily dosing, which is particularly suitable for elderly patients with venous thromboembolism. On postoperative day seven, lower extremity venous ultrasound confirmed continued graft patency, and the patient was discharged on postoperative day nine.

**Figure 6:**
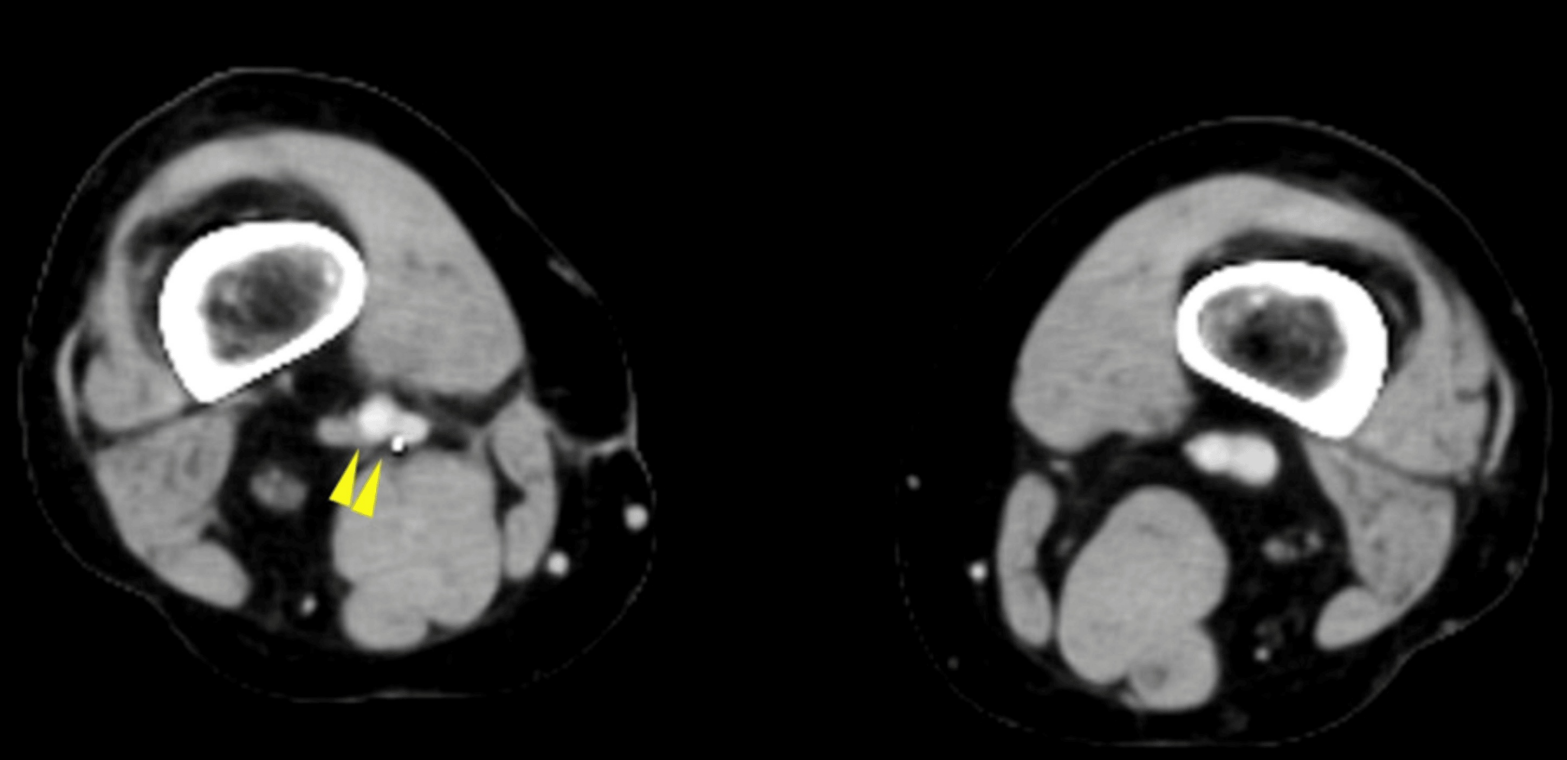
Contrast-enhanced CT at 1.5 years postoperatively. The venous graft remained patent without evidence of stenosis or thrombus (yellow arrowheads). Images were obtained using a 64-slice multidetector CT scanner.

A surveillance protocol was implemented with duplex ultrasound examinations every three months during the first postoperative year and then annually. At 1.5 years post-surgery, contrast-enhanced CT demonstrated sustained patency of the venous graft (Figure 6), and the patient remained asymptomatic without leg-related complications.

## Discussion

PVAs, though rare, with reported incidence rates ranging from 0.2% to 0.28% in patients undergoing imaging for venous disorders, present significant clinical challenges due to their potential for severe thromboembolic complications, most notably PE [1]. In adults, the typical normal diameter of the popliteal vein is approximately 5-7 mm. Aneurysms exceeding twice this diameter are generally considered clinically significant, while those surpassing threefold dilation are particularly high-risk for embolic events. Notably, PE occurs in approximately 39%-71% of PVA cases, underscoring the insufficiency of anticoagulation alone, as recurrence rates in anticoagulated patients without surgical intervention may reach nearly 80% [2]. Consequently, surgical intervention remains the cornerstone of management to prevent recurrent thromboembolic events, particularly in symptomatic cases or those with substantial aneurysmal dilatation or complex morphology [3]. Our patient, a 77-year-old woman who presented with symptomatic PE secondary to a significantly thrombosed PVA nearly three times the normal diameter, met clear indications for surgical treatment.

While tangential aneurysmectomy remains the most commonly performed technique due to its relative simplicity and well-documented outcomes, its efficacy may be compromised in cases with extensive thrombus burden, as incomplete thrombus removal can lead to recurrence. Moreover, recurrence rates following tangential aneurysmectomy have been reported to range from 10% to 30%, particularly in cases with complex morphology or residual thrombus [3,4]. In our case, the aneurysm exhibited a substantial thrombotic load adherent to the vessel wall, necessitating complete aneurysmal resection. Furthermore, the notable diameter discrepancy between the native popliteal vein and the GSV graft required meticulous graft construction. Several techniques, including the autologous saphenous vein panel graft (ASVPG), which provides a flat, widened lumen, and the spiral graft, which offers greater length and flexibility, have been described for such reconstructions [5,6]. Compared to these, the roll graft technique allowed for more controlled tailoring of the graft diameter, a simpler construction method, and preservation of longitudinal venous flow characteristics, making it particularly suitable for this patient with moderate size mismatch and limited operative field.

In this patient, the proximal and distal segments of the popliteal vein measured approximately 6 mm in diameter, whereas the harvested GSV measured approximately 3 mm - nearly half the size. To address this disparity, we harvested a GSV graft twice the length of the aneurysm, divided it longitudinally, and meticulously sutured the long edges together to construct a customized roll graft tailored to the patient's anatomical requirements.

This approach offered several advantages, primarily preserving the autologous tissue properties known for superior biocompatibility, lower infection risk, and enhanced resistance to thrombosis compared to synthetic graft materials. Additionally, fabricating a graft of optimal dimensions allowed for improved hemodynamic flow characteristics, potentially mitigating the risk of future thrombotic events. Notably, harvesting the contralateral GSV preserved the ipsilateral vein, safeguarding alternative venous conduits for potential future interventions. Nevertheless, potential long-term concerns with roll graft reconstruction include the risk of neointimal hyperplasia, graft stenosis, or venous insufficiency, particularly in high-flow settings. Regular postoperative surveillance through duplex ultrasonography is therefore recommended.

Postoperative anticoagulation strategies in venous aneurysm cases, including post-surgical patients, traditionally advocate for warfarin therapy over a period of three to six months. However, evolving clinical evidence has increasingly favored direct oral anticoagulants (DOACs) due to their ease of administration and predictable pharmacokinetics. Reflecting this shift in practice, we employed a short initial course of systemic heparin therapy, followed by a transition to DOAC therapy, with sustained efficacy demonstrated by long-term graft patency and the absence of thromboembolic recurrence at 1.5 years post-surgery. Although lifelong anticoagulation is not universally mandated, extended therapy may be considered in high-risk patients. In our case, the patient remained on DOACs beyond one year, with periodic follow-up including duplex ultrasonography every six months to monitor graft patency and thrombotic risk.

## Conclusions

This case report highlights that resection of a thrombosed popliteal venous aneurysm, coupled with a customized great saphenous vein roll interposition graft, can achieve successful restoration of venous flow and durable mid-term graft patency. While roll graft techniques have been described in other vascular contexts, their application in popliteal venous aneurysms remains relatively uncommon. Our follow-up protocol included duplex ultrasonography at six-month intervals up to one year, followed by contrast-enhanced CT at 1-1.5 years postoperatively, all of which demonstrated sustained graft patency and the absence of thromboembolic complications. Nonetheless, the lack of comparative data and the limited follow-up beyond 1.5 years preclude definitive conclusions regarding the long-term efficacy and potential complications. Moreover, although the roll graft was feasible and provided an optimal diameter match in this patient, its applicability may vary across different patient populations depending on venous diameter, comorbidities, and the availability of adequate saphenous vein. While our findings suggest promising outcomes, further case accumulation and comparative studies are necessary to determine whether this approach is clearly superior to conventional techniques, elucidate long-term thrombosis risk, refine anticoagulation strategies, and establish patient selection criteria.
